# Sequential Dosing in Chemosensitization: Targeting the PI3K/Akt/mTOR Pathway in Neuroblastoma

**DOI:** 10.1371/journal.pone.0083128

**Published:** 2013-12-31

**Authors:** Mike-Andrew Westhoff, Najmeh Faham, Daniela Marx, Lisa Nonnenmacher, Claudia Jennewein, Stefanie Enzenmüller, Patrick Gonzalez, Simone Fulda, Klaus-Michael Debatin

**Affiliations:** 1 Department of Pediatrics and Adolescent Medicine, University Medical Center Ulm, Ulm, Germany; University of Quebec at Trois-Rivieres, Canada

## Abstract

Breaking resistance to chemotherapy is a major goal of combination therapy in many tumors, including advanced neuroblastoma. We recently demonstrated that increased activity of the PI3K/Akt network is associated with poor prognosis, thus providing an ideal target for chemosensitization. Here we show that targeted therapy using the PI3K/mTOR inhibitor NVP-BEZ235 significantly enhances doxorubicin-induced apoptosis in neuroblastoma cells. Importantly, this increase in apoptosis was dependent on scheduling: while pretreatment with the inhibitor reduced doxorubicin-induced apoptosis, the sensitizing effect in co-treatment could further be increased by delayed addition of the inhibitor post chemotherapy. Desensitization for doxorubicin-induced apoptosis seemed to be mediated by a combination of cell cycle-arrest and autophagy induction, whereas sensitization was found to occur at the level of mitochondria within one hour of NVP-BEZ235 posttreatment, leading to a rapid loss of mitochondrial membrane potential with subsequent cytochrome *c* release and caspase-3 activation. Within the relevant time span we observed marked alterations in a ∼30 kDa protein associated with mitochondrial proteins and identified it as VDAC1/Porin protein, an integral part of the mitochondrial permeability transition pore complex. VDAC1 is negatively regulated by the PI3K/Akt pathway via GSK3β and inhibition of GSK3β, which is activated when Akt is blocked, ablated the sensitizing effect of NVP-BEZ235 posttreatment. Our findings show that cancer cells can be sensitized for chemotherapy induced cell death – at least in part – by NVP-BEZ235-mediated modulation of VDAC1. More generally, we show data that suggest that sequential dosing, in particular when multiple inhibitors of a single pathway are used in the optimal sequence, has important implications for the general design of combination therapies involving molecular targeted approaches towards the PI3K/Akt/mTOR signaling network.

## Introduction

Neuroblastoma (NB) is a common childhood neoplasia of the sympathetic nervous system that presents as a highly heterogeneous disease, ranging from spontaneous regression to high risk of fatality due to multimodal therapy resistance [1], [2]. The advanced stages of this malignancy are difficult to treat and despite intense therapeutic intervention the cure rates for high grade NB have only improved marginally over the recent years [3].

We previously found that phosphorylated Akt correlates with poor patients' prognosis in NB [4], and the PI3K/Akt pathway has subsequently been linked to augmented cell survival [5] and increased resistance to chemotherapy in this tumor [6]. The potential of NVP-BEZ235, a novel PI3K/mTOR inhibitor, as a single therapeutic agent has already been investigated in MYCN-amplified neuroblastoma, where it could be shown to exert both, an antiproliferative effect and a blockage of tumor angiogenesis [7]. The same work further suggests that monotherapy consisting of PI3K/mTOR inhibition alone is ineffective in neuroblastoma that do not harbor a MYCN amplification [7], which led us to speculate that NVP-BEZ235 might be better suited as part of a targeted combination therapy. This is of particular interest, as inhibition of PI3K/Akt mediated signaling strongly amplifies cell death induced by a wide range of chemotherapeutics [8]. The aim of combining pharmacological inhibitors of cell signaling (sensitizers) – such as NVP-BEZ235 – with more conventional chemotherapy (inducers) is to enhance tumor-specific cell death, while concomitantly reducing side effects.

Since therapy resistance appears to be a key feature of many tumors, including advanced neuroblastoma [3], breaking this resistance is a major goal in the development of novel therapeutic approaches. Given the fact that elimination of tumor cells requires induction of cell death pathways, which may be counteracted by increased activity of survival signaling, targeting survival pathways such as the PI3K/Akt-signaling network by appropriate inhibitors appears to be a promising strategy for overcoming therapy resistance [9]. While this signaling cascade has long since been proposed to be a opportune target in cancer therapy and several clinical trials are ongoing, the promising experimental results so far have not been translated into therapeutic successes. Currently, only inhibitors of mTOR are approved for cancer therapy [10]. While the success of targeting PI3K/Akt can doubtless be potentiated by improved use of predictive biomarker strategies [11], certain unpredicted features of pharmacological PI3K/mTOR inhibitors have emerged that suggest it is important to reevaluate the protocols of how these substances are best applied. For example, recent data suggest that GDC-0941, a potent PI3K inhibitor, can alter tumor microvascularisation and thus, depending on tumor type, enhance or reduce the amount of chemotherapeutic agent and inhibitor which is subsequently delivered to the tumor [12, Nonnenmacher unpublished data]. Therefore, in contrast to conventional chemotherapy, targeted therapy affects specific signaling pathways that may synergize with, or antagonize conventional chemotherapy and thus targeting survival pathways may require a more in depth understanding of sequential dosing, i. e. downregulation of survival signaling before, during or after addition of chemotherapy may lead to completely different results. Interestingly, scheduling in the combination of apoptosis sensitizers and apoptosis inducers has only recently come into the focus of scientific investigations [13]. Novel data suggest that optimum sensitization for apoptosis may not require prolonged full inhibition of survival pathways [14].

To elucidate this neglected aspect of combination therapy, i. e. the role of sequential scheduling of inducer and sensitizers, we used several inhibitors of the PI3K/Akt network, such as the pan-specific PI3K-inhibitor NVP-BEZ235, in combination with doxorubicin, a drug frequently used in NB treatment [15].

## Materials and Methods

### Cell culture

SHEP NB and D54 GBM cells were maintained in DMEM, while SH-SY5Y NB and Kelly NB were cultured in RPMI 1640 (both Gibco® Life Technologies, Darmstadt, Germany). Media were supplemented with 10% FCS (Biochrom, Berlin, Germany), 1 mmol/L glutamine (Biochrom) and 1% penicillin/streptomycin (Biochrom).

All cell lines were obtained from ATCC.

### Pharmacological inhibitors

0.6 µM NVP-BEZ235 (Novartis Pharma GmbH, Basel, Switzerland)

10 µM SB415286 (Tocris Bioscience, Mi, USA)

0.6 µM PI-103 (Cayman Chemical, Mi, USA)

20 nM Bafilomycin A1 (Sigma-Aldrich, Munich, Germany)

0.6 µM NVP-BKM120 (Novartis Pharma GmbH, Basel, Switzerland)

0.1 µM rapamycin (Sigma-Aldrich)

10 µM Nu7026 (Sigma-Aldrich)

### Protein immunoblotting

Western blot analysis was carried out as previously described [16], using the following antibodies: rabbit polyclonal caspase-3 (Cell Signaling, Beverly, MA, USA), mouse monoclonal β-actin (Sigma-Aldrich, Munich, Germany), rabbit polyclonal phosphoserine/threonine antibody (Abcam, Cambridge, UK) mouse monoclonal Akt (BD Bioscience, Heidelberg, Germany), rabbit polyclonal phospho-Akt (Ser473), rabbit polyclonal phospho-S6 ribosomal protein (Ser235/236), rabbit anti-S6 ribosomal protein (all Cell Signaling, Beverly, MA, USA), rabbit polyclonal anti-LC3 (Thermo Fisher Scientific, Braunschweig, Germany), rabbit polyclonal phospho-GSK3α/β (Cell Signaling), mouse monoclonal GAPDH (HyTest, Turku, Finland) followed by anti-mouse or anti-rabbit immunoglobulin G–horseradish peroxidase from Santa Cruz Biotechnology (Santa Cruz Biotechnology, CA, USA). Enhanced chemiluminescence was used for detection (Amersham Bioscience, Freiburg, Germany).

### Cell death measurement

The cell death readout was DNA fragmentation (sub-G1 peak), a hallmark of apoptosis, as assessed by fluorescence-activated cell-sorting (FACScan, BD Bioscience) analysis of DNA fragmentation of propidium iodide-stained nuclei as previously described [17]. The specific sub-G1 peak was calculated as follows: 100×(experimental DNA fragmentation (%)−spontaneous DNA fragmentation (%))/(100%−spontaneous DNA fragmentation (%)).

### Cell counting

Cells were seeded and allowed to proliferate for indicated periods of time. This was followed by prolonged treatment with a trypsin/EDTA solution (Biochrom) until all cells were in suspension. Cell suspension was diluted 1∶100 in CASYton solution (Innovatis, Reutlingen, Germany) and cell number was then determined using CASY1 DT (Innovatis).

### Cell cycle distribution

The cell cycle distribution was assayed similarly to apoptosis, as described above. The distribution of 20,000 cells was then determined by the Dean Jet Fox Model using the *Flow Jo* software (Tree Star, Inc., Standford, CA, USA).

### Ki67 expression

Cells were treated and analyzed as previously described [17]. Staining was performed with 1∶100 dilutions of anti-Ki67 antibody (AbD Serotec, Düsseldorf, Germany) and a FITC-labeled secondary antibody (Millipore, Billerica, MA, USA). The proliferation index was defined as percentage of Ki67 positive cells within a population. Per experiment, a minimum of 100 cells was assessed.

### Chemotherapeutica

Doxorubicin: Adrimedac® (Pharmachemie B. V., Haarlem, Netherlands)

Cisplatin (Teva Pharmaceutical Industries, Petah Tikva, Israel)

Topotecan: Hycamtin® (GlaxoSmithKline, London, UK)

Etoposide: Etopophos® (Bristol-Myers Squibb, New York, NY, USA)

### Cytochrome c release

Cytochrome *c* release was determined by fluorescence-activated cell-sorting as previously described [16], using a 1∶20 dilution of anti-cytochrome *c* antibody (BD Bioscience) followed by an additional wash and incubation with a FITC-labeled secondary antibody (Santa Cruz Biotechnology). The increase of cytosolic cytochrome *c*, which cannot be detected by this specific antibody, was assessed as decrease of fluorescence.

### Loss of mitochondrial membrane potential measurement

A stock solution of 20 mg TMRM (Sigma-Aldrich) per ml DMSO was diluted 1∶20,000 in complete medium and then used at a 1∶10 dilution for 10 min at 37°C. After medium removal cells were treated with trypsin and resuspended in PBS prior to fluorescence-activated cell-sorting.

### Single-cell gel electrophoresis (Comet) assay

DNA damage was assayed by alkaline comet assay, as previously described [17]. A total of 100 randomly selected cells per experiment were measured by image analysis (Kinetic Imaging Komet 5.0 Software, Andor Technology Ltd, Berlin, Germany) using an Olympus AX70 ‘Provis’ microscope (Hamburg, Germany). DNA damage is expressed as Mean Olive Tail Moment.

### Determination of autophagy

Cell were treated and fixed/stained at the indicated time points using aforementioned LC3 antibody and FITC-labeled secondary antibody. Percentage of autophagic cells was calculated by dividing the number of cells with clear punctuate staining (indicative of autophagosomes) through total cell number.

### Protein co-immunoprecipitation

Cells were lysed for 15 min in RIPA buffer (100 mM Tris-HCl pH 7.4, 300 mM NaCl, 2% NP-40, 1% Sodium Deoxycholate, 0.2% SDS, all Sigma-Aldrich) in the presence of the same inhibitor cocktail used for protein immunoblotting. After centrifugation for 15 min at 14,000 rpm and 4°C, following antibodies were added for o/n incubation under agitation at 4°C: Either 1∶200 rabbit polyclonal Bim (Cell Signaling), 1∶50 rabbit polyclonal Bad (Cell Signaling), or 1∶50 mouse monoclonal Bax (BD Biosciences) in combination with 1∶50 rabbit polyclonal Bax NT (Millipore), or 1∶100 rabbit polyclonal VDAC1/Porin (Abcam) or 1∶100 mouse GSK3β (BD Biosciences). 50 µl Protein G-Agarose was added for 2 hrs, followed by three washes with PBS. Samples were then prepared for immunoblotting.

### Colony forming assay

Following treatment cells were diluted 120-fold and allowed to grow for 10 days, then washed in PBS, fixed in 3.7% paraformaldehyde for 15 min and stained in a 1∶10 Giemsa/water mix for 10 min. After several washes with water, cells were allowed to dry.

## Results and Discussion

### Characterization of NVP-BEZ235 as a single agent

**Figure 1:**
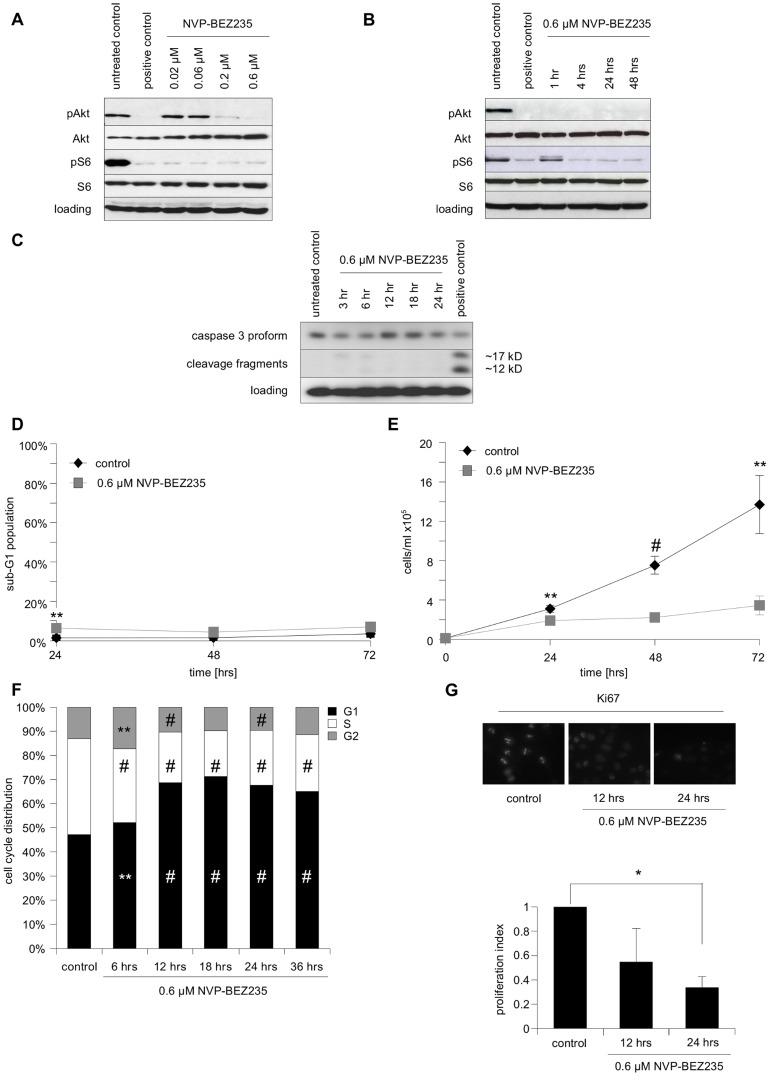
The effects of the PI3K/mTOR inhibitor NVP-BEZ235 on SHEP NB cells. **A** Cells were either left untreated, treated for 24 µM PI-103, a well-characterized pan-PI3K inhibitor used as positive control, or the indicated concentrations of NVP-BEZ235. Protein expression levels and phosphorylation status of Akt and S6 ribosomal protein served as surrogate read-outs for PI3K and mTOR activity, respectively, and were analyzed by Western blotting, β-actin served as loading control. **B** Cells were either left untreated, treated for 24 hrs with either 0.6 µM PI-103 as positive control, or 0.6 µM NVP-BEZ235 for the indicated lengths of time. Protein expression levels and phosphorylation status of Akt and S6 ribosomal protein were analyzed by Western blotting, β-actin served as loading control. **C** Cells were either left untreated, treated for indicated length of time with 0.6 µM NVP-BEZ235, or treated for 24 hrs with 0.2 µg/ml doxorubicin as positive control. Protein expression levels and cleavage of caspase-.3 protein were analyzed by Western blotting, β-actin served as loading control. **D** Cells were cultured either in the presence or absence of 0.6 µM of NVP-BEZ235 for 24, 48 and 72 hrs, followed by FACS analysis of the DNA fragmentation of propidium iodide-stained nuclei. The percentage of absolute DNA fragmentation is shown as readout of apoptosis. **E** 24, 48 and 72 hrs after treatment with 0.6 µM NVP-BEZ235 total cell numbers of treated and untreated cells were counted. **F** Cell cycle distribution (untreated control and samples treated with 0.6 µM of NVP-BEZ235) was determined after indicated times by FACS analysis of propidium iodide-stained nuclei. **G** Either untreated controls, or cells treated for 12 and 24 hrs with 0.6 µM NVP-BEZ235 were stained for Ki67 protein expression and evaluated by immunofluorescent microscopy. In A–C and F a representative result of two independent experiments is depicted, while in D and E mean+s.e.m. values of at least three independent experiments carried out in triplicate are shown. Shown in F is the mean of three independent experiments carried out in triplicate, in G the mean+SD of three independent experiments. Statistical analysis was carried out by two-sided Student's *t*-test; * P-value <0.01; ** P-value <0.001; # P-value <0.0001.

First, we characterized the effect of the dual kinase inhibitor NVP-BEZ235, which blocks PI3K and mTOR activity, on SHEP NB cells, using phosphorylation of Akt and S6 as surrogate read-outs (Fig. 1A). A concentration of 0.6 µM NVP-BEZ235 was shown to lead to a rapid and prolonged inhibition of the PI3K/Akt signaling cascade (Fig. 1B). This concentration exerted little toxic effect, as indicated by the absence of both, caspase-3 processing (Fig. 1C) and strong, prolonged DNA fragmentation (Fig. 1D). In contrast, 0.6 µM NVP-BEZ235 already profoundly affected the cell number of treated cells, almost completely blocking any increase of cell numbers over a time span of at least 72 hrs (Fig. 1E). The stasis in cell numbers is most likely due to a block in proliferation, as indicated by a marked G_1_/G_0_ arrest in the cell cycle (Fig. 1F) and a significant decrease in Ki67 positive cells within 24 hrs (Fig. 1G). This seems to be mainly mediated by PI3K inhibition, as a related compound, NVP-BKM120, which only targets PI3K, had similar effects on cell numbers and cell cycle distribution (Fig. S1), while rapamycin, which only inhibits mTOR, had a much less pronounced effect (Fig. S2).

Taken together, these findings indicate that SHEP NB cells treated with subtoxic concentrations of NVP-BEZ235 temporarily exit the cell cycle due to reduced PI3K signaling and, thus, stop dividing.

### NVP-BEZ235 and Doxorubicin in combination therapy

To determine how best to combine NVP-BEZ235 and doxorubicin to maximize therapeutic efficacy, we next elucidated the effects of doxorubicin alone. Treatment of SHEP NB cells with 0.2 µg/ml of doxorubicin led to alterations in the cell cycle within 6 hours, although a significant G_2_ arrest only arose after 18 hrs (Fig. S3A). Cleavage of caspase-3, used as a surrogate read-out for the cells' irrevocable commitment to apoptosis, was detectable after 15 hrs (Fig. S3B) and no DNA fragmentation was detectable for at least up to 12 hours after treatment (Fig. S3C). We further verified that doxorubicin-induced apoptosis is independent of the population's cell cycle distribution (Fig. S3D) and, thus, that the G_1_/G_0_ arrest promoted by NVP-BEZ235 should not interfere with the cytotoxic effect of the chemotherapeutic agent.

**Figure 2:**
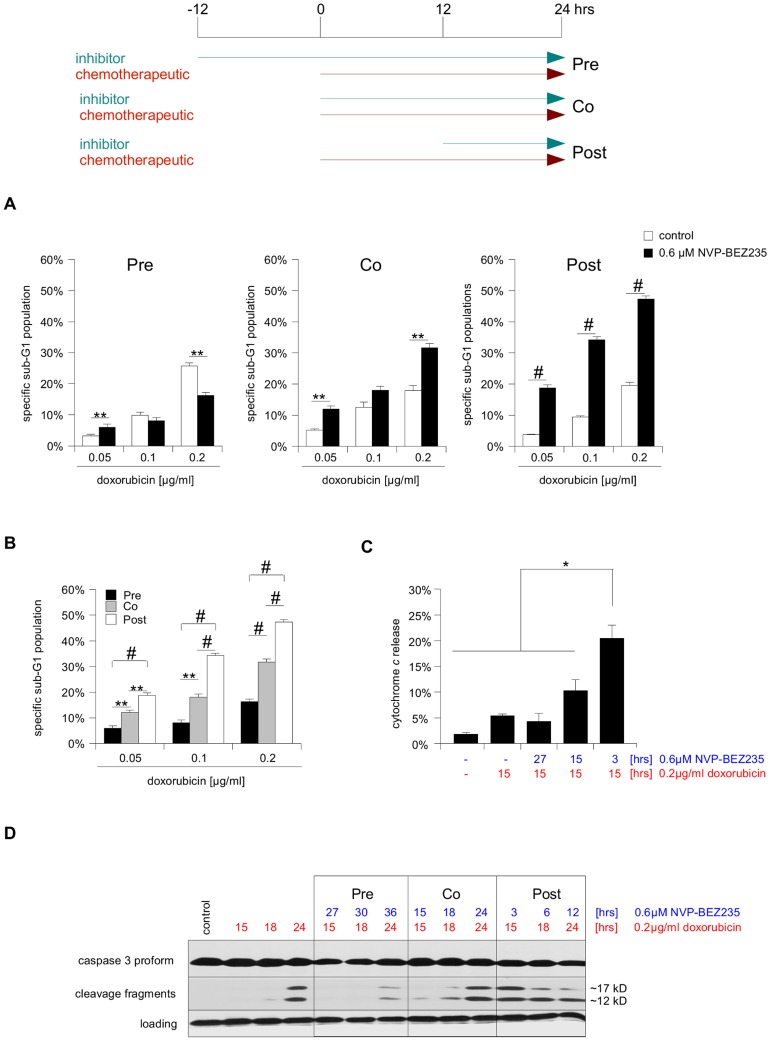
Altered timing affects the potency of NVP-BEZ235/doxorubicin combination therapy in SHEP NB cells. Three different treatment combinations were tested on SHEP NB cells, giving NVP-BEZ235 12 hrs prior to doxorubicin (Pre), giving both substances concurrently (Co), or giving NVP-BEZ235 12 hrs after the chemotherapeutic (Post). Importantly, the maximal incubation time with doxorubicin was kept constant at 24 hrs (earlier time points also shown in C and D). **A** SHEP NB cells were treated with NVP-BEZ235 and indicated concentrations of doxorubicin for 24 hrs, according to the scheme outlined above. Apoptosis was determined by FACS analysis of the DNA fragmentation of propidium iodide-stained nuclei, and percentage of specific DNA fragmentation is shown. **B** An alternative depiction of the data presented in A, highlighting the difference between the three NVP-BEZ235/doxorubicin combinations. For all following experiments 0.2 µg/ml doxorubicin was used. **C** Cells were either left untreated or treated as indicated and mitochondrial release of immunofluorescent-labeled cytochrome c was determined by FACS analysis. **D** Cells were either left untreated or treated as indicated. A Western blot analysis of caspase-3 processing served as surrogate read-out of caspase activation (appearance of the ∼12 kD cleavage fragment), β-actin was used as loading control. In A and B mean+s.e.m. values of three independent experiments carried out in triplicate, in C mean+s.d. of three independent experiments are shown, while in D a representative result of three independent experiments is depicted. Statistical analysis was carried out by two-sided Student's *t*-test; * P-value <0.01; ** P-value <0.001; # P-value <0.0001.

Combining these data sets of the two individual substances, we devised the treatment schedule shown in Figure 2: SHEP NB cells were either treated for 12 hrs with NVP-BEZ235 prior to the addition of doxorubicin for 24 hrs (pretreatment), or NVP-BEZ235 and doxorubicin were given concurrently for 24 hrs (cotreatment), or NVP-BEZ235 was only added for the last 12 hrs of a 24 hrs doxorubicin treatment (posttreatment). It is important to note that the treatment time with the chemotherapeutic remained constant at 24 hrs for all three different schedules.

Interestingly, these schedules had distinct and significantly different outcomes on apoptosis induction. While no positive sensitization effect for pretreatment could be observed compared to doxorubicin alone, cotreatment in contrast led to a moderate increase in cell death at most concentrations, while posttreatment led to the strongest and most consistent apoptosis sensitization (Fig. 2A). Comparing the three schedules directly with each other, one can clearly observe a continuous improvement from pre- to co- to posttreatment (Fig. 2B). These differences in cell death are also reflected in the levels of cytochrome *c* release from the mitochondria (Fig. 2C) and cleavage of caspase-3 (Fig. 2D).

**Figure 3:**
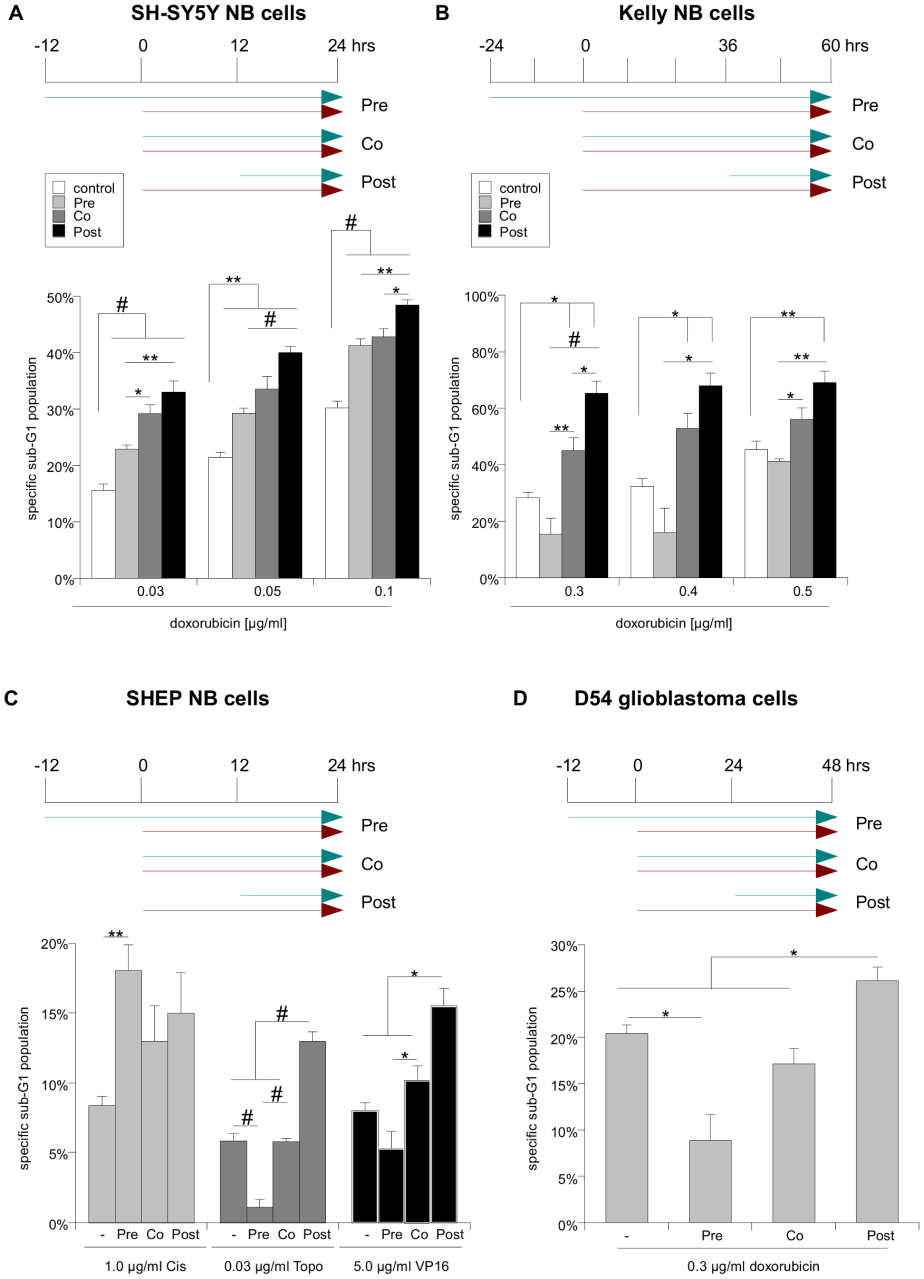
The superiority of posttreatment with NVP-BEZ235 is not restricted to one NB cell line and doxorubicin. **A** SH-SY5Y NB cells were treated as indicated by the scheme and apoptosis was determined by FACS analysis of the DNA fragmentation of propidium iodide-stained nuclei. **B** Kelly NB cells were treated as indicated by the scheme and apoptosis was determined by FACS analysis of the DNA fragmentation of propidium iodide-stained nuclei. **C** SHEP NB cells were treated as indicated by the scheme, substituting doxorubicin with either 1.0 µg/ml Cisplatin (Cis), 0.03 µg/ml Topotecan (Topo) or 5.0 µg/ml Etoposide (VP16). Apoptosis was determined by FACS analysis of the DNA fragmentation of propidium iodide-stained nuclei. **D** D54 glioblastoma cells were treated as indicated by the scheme and apoptosis after doxorubicin (0.3 µg/ml doxorubicin) treatment was determined by FACS analysis of the DNA fragmentation of propidium iodide-stained nuclei. In A to D mean+s.e.m. values of three independent experiments carried out in triplicate are shown. Statistical analysis was carried out by two-sided Student's *t*-test; * P-value <0.01; ** P-value <0.001; # P-value <0.0001.

We next confirmed the validity of our findings using two additional cell lines, SH-SY5Y and Kelly NB cells (3A and B, respectively). These cell lines differ in *MYCN* status (Kelly: amplified, SH-SY5Y: non-amplified; [18]) and 11q deletion (Kelly: deleted, SH-SY5Y: not deleted; [19]), also SH-SY5Y NB cells are 1q trisomic [20], while this particular chromosomal abnormality is not found in Kelly NB cells [21]. Thus, these two cell lines can be viewed as representing different extremes on the spectrum of malignant NB [22]. In addition, we also used three additional drugs commonly used in NB treatment, Cisplatin, Topotecan, Etoposide, and could show a superior sensitizing effect of posttreatment with NVP-BEZ235 when combined with two of these drugs, Topotecan and Etoposide (Fig. 3C). Furthermore, we used the glioblastoma (GBM) cell line D54 to confirm that the observed efficacy of posttreatment is not confined to NB cells (Fig. 3D). Here, we could also observe increased apoptosis sensitivity to doxorubicin-induced cell death when NVP-BEZ235 was added 12 hrs after the chemotherapy.

Taken together these data are consistent with the hypothesis that blocking the PI3K/Akt signaling cascade can sensitize several cancer cell lines for apoptosis induced by DNA-damaging drugs. Importantly, a prolonged inhibition is less potent – and frequently even causes an apoptosis desensitization – than an inhibition of PI3K/Akt signaling that is only initiated after extended application of chemotherapeutic agents.

### Molecular mechanism of apoptosis sensitization

**Figure 4:**
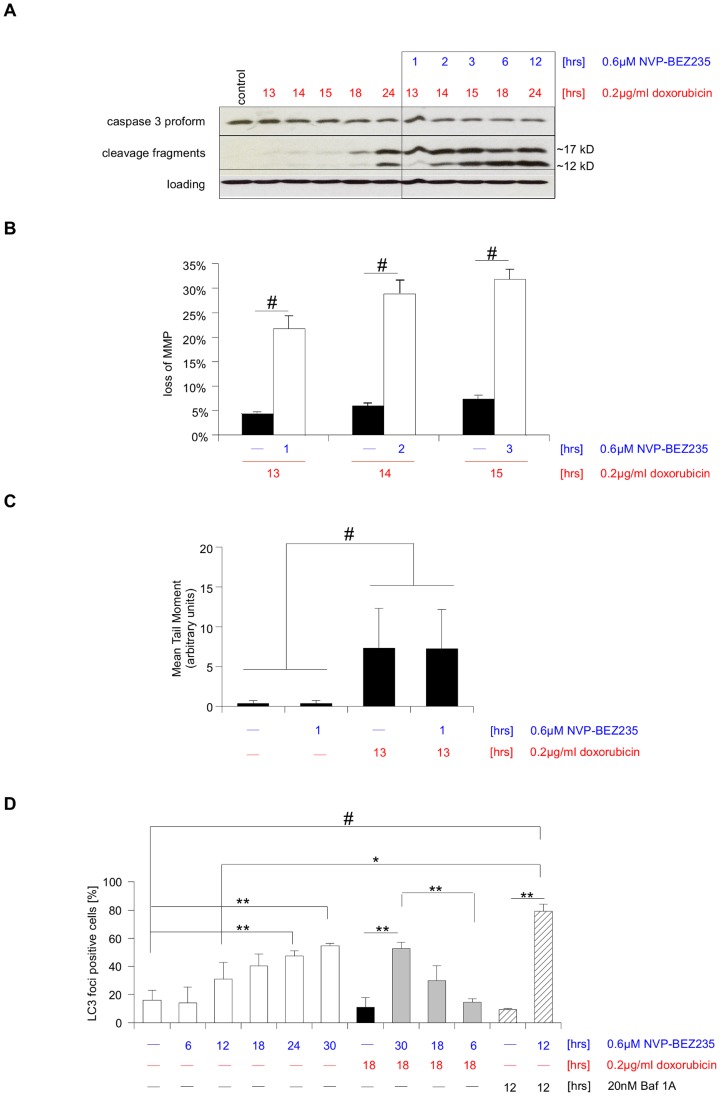
Apoptosis sensitization occurs at the mitochondrial level. **A** SHEP NB cells were either left untreated (control) or treated as indicated, followed by a Western blot analysis of the caspase-3 processing kinetic, with β-actin as loading control. **B** The loss of mitochondrial membrane potential (MMP) was analyzed after indicated treatment. Cells were incubated with TMRM dye prior to FACS analysis. **C** Cells were either left untreated or treated as indicated. The DNA damage was assayed by single cell gel electrophoresis (Comet) assay and expressed as Mean Olive Tail Moment. **D** Cells were treated for the indicated length of time with 0.6 µM NVP-BEZ235, 0.2 µg/ml doxorubicin, 20 nM Bafilomycin A1 (a inhibitor of the late stages of autophagy that blocks fusion between autophagosomes and lysosomes), or combinations thereof. The percentage of autophagic cells was then determined by counting cells with LC3 foci. In A representative results of two independent experiments are shown, in B mean+s.e.m. of three independent experiments carried out in triplicate are shown, while in C mean+s.d. of two independent experiments are depicted. In D mean+s.d. Of three independent experiments are depicted. Statistical analysis was carried out by two-sided Student's *t*-test; * P-value <0.01; ** P-value <0.001; # P-value <0.0001.

Next, we determined at which level of the apoptotic signaling cascade posttreatment-mediated sensitization occurs. As NVP-BEZ235 single treatment does not lead to caspase-3 cleavage, while doxorubicin treatment does (Fig. 1C), we initially concentrated on caspase activation (Fig. 4A). Differences in caspase-3 cleavage (Fig. 4A), as well as loss of mitochondrial membrane potential (Fig. 4B) between posttreatment and treatment with doxorubicin alone were already clearly visible within less than 1 hour of NVP-BEZ235 addition. As no differences in DNA damage/repair could be detected within a similar time span (Fig. 4C), we concluded that the sensitization effect of NVP-BEZ235 most likely occurs after induction of DNA damage, but prior to destruction of the mitochondria. As not only apoptosis resistance, but several other cellular aspects, such as lysosome biogenesis and autophagy [23], [24], [25], have been described to be affected by modulation of PI3K signaling [26] we also investigated the role of autophagy in our experimental setting. This is of particular interest as autophagy has been associated with both sensitization for [25], [27] and protection from cell death [28]. Our data show that LC3 conversion only occurs after approximately 12 hrs (Fig. S3E), i.e. only at the very end of posttreatment. In addition, we investigated the appearance of LC3 foci, indicative of autophagosomes formation (Fig. 4D). A fully balanced autophagic flux relies on autophagosome formation and autophagosomal-lysosomal fusion events, leading to the degradation of cellular cargo. The integrity of this process can be assayed in the presence of Bafilomycin A1, a substance that blocks autophagolysosome formation by inhibiting autophagosomal-lysosomal fusion. The combination of NVP-BEZ235 and BafilomycinA1 strongly increased the amount of LC3 positive cells compared to NVP-BEZ235 treatment alone, indicating that treatment with NVP-BEZ235 efficiently triggers autophagy induction (Fig. 4D). Interestingly, a comparison of the different treatment schedules suggests that an increase in autophagy correlates with reduced apoptosis (Fig. 4D).

**Figure 5:**
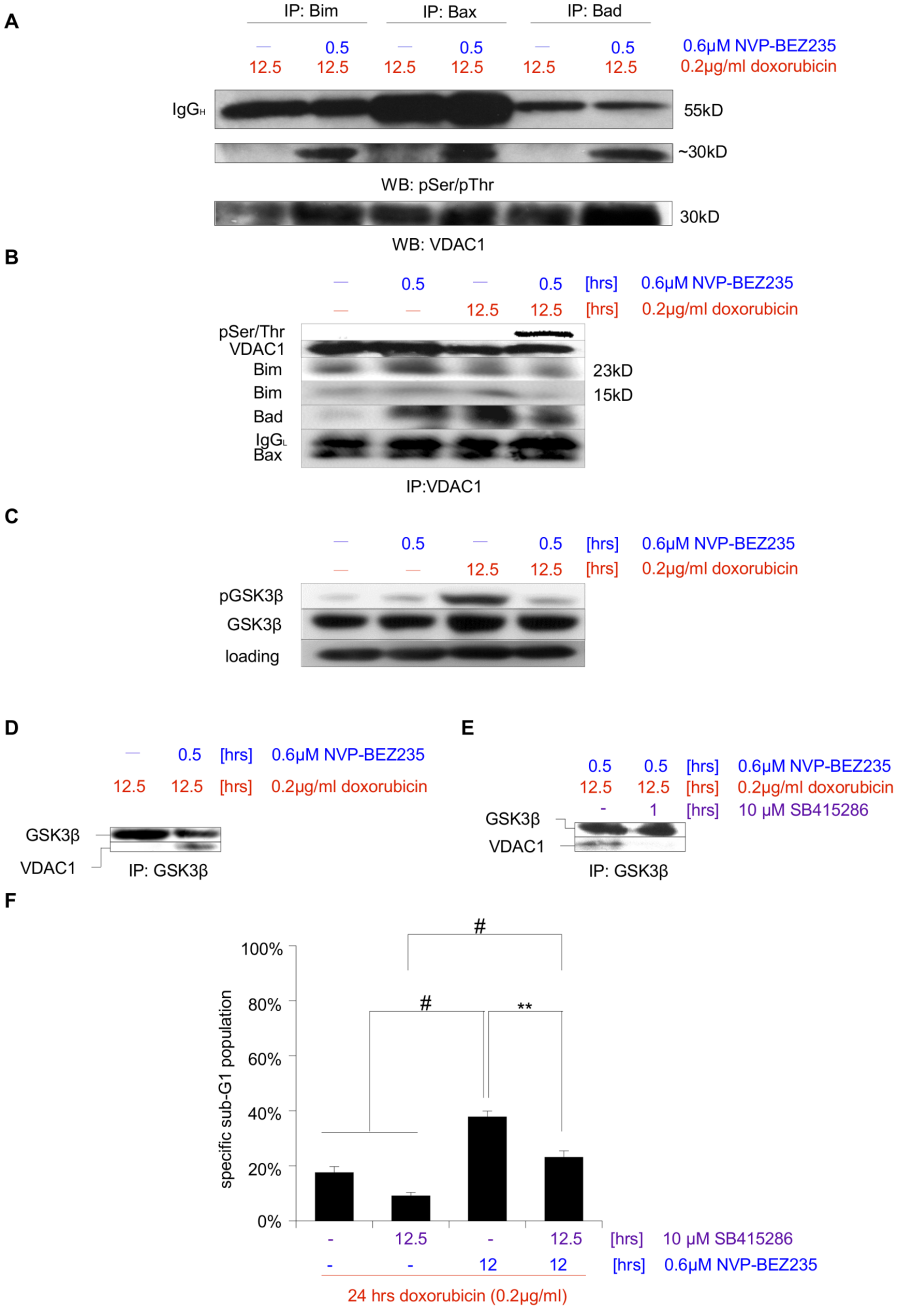
Sensitization for doxorubicin-induced apoptosis via posttreatment with NVP-BEZ235 is mediated via VDAC1. **A** SHEP NB cells were treated for 12.5-BEZ235 for the last 0.5 hr. Either Bim, Bax or Bad was then immunoprecipitated and interaction partners that are phosphorylated on Serine or Threonine were visualized by Western blot analysis. A ∼30 kD protein, the presence of which appears to depend on NVP-BEZ235 addition, was identified as VDAC by VDAC1/Porin-specific antibody. IgG_H_ – heavy chain. **B** Cells were left untreated, treated for 12.5 hrs with Doxorubicin, or after 12 hrs for 0.5 hr with NVP-BEZ235, or a combination of both (first 12 hrs with Doxorubicin alone, followed by the addition of NVP-BEZ235 for 0.5 hr). VDAC was immunoprecipitated and its phosphorylation status was probed. IgG_L_ – light chain. **C** Cells were left untreated, treated for 12.5 hrs with doxorubicin, or after 12 hrs for 0.5 hr with NVP-BEZ235, or a combination of both (first 12 hrs with doxorubicin alone, followed by the addition of NVP-BEZ235 for 0.5 hr). Protein expression levels and phosphorylation status of GSK3β were analyzed by Western blotting, GAPDH served as loading control. **D** Cells were treated either for 12.5 hrs with doxorubicin, or a combination of doxorubicin and NVP-BEZ235, (first 12 hrs with doxorubicin alone, followed by the addition of NVP-BEZ235 for 0.5 hr). This was followed by immunoprecipitation of GSK3β and analysis of this protein's interaction with VDAC via immunoblotting. **E** Cells were again treated with a combination of doxorubicin and NVP-BEZ235, (first 12 hrs with doxorubicin alone, followed by the addition of NVP-BEZ235 for 0.5 hr), during the last hour in the absence or presence of the GSK3β-specific inhibitor SB415286. This was followed by immunoprecipitation of GSK3β and analysis of this protein's interaction with VDAC. **F** Apoptosis in cells treated for 24 hrs with doxorubicin, for 12.5 hrs with SB415286, for 12 hrs with NVP-BEZ235, or a combination of those substances was determined by FACS analysis of the DNA fragmentation of propidium iodide-stained nuclei, and percentage of specific DNA fragmentation is shown. Shown in A to E are representative blots of at least two independent experiments, in F the mean+s.e.m. of three independent experiments performed in triplicate is depicted. Statistical analysis was carried out by two-sided Student's *t*-test; * P-value <0.01; ** P-value <0.001; # P-value <0.0001.

As NVP-BEZ235 itself causes neither caspase activation (Fig. 1), nor DNA damage (Fig. 4C), it most likely causes increased apoptosis upon doxorubicin treatment by enhancing the efficacy of chemotherapy. The short time span during posttreatment from NVP-BEZ235 addition to the cells' irrevocable commitment to apoptosis strongly argues for a sensitization effect mediated by phosphorylation of proteins associated with the mitochondria. For a translational/transcriptional event to contribute significantly, alterations at the mitochondria occur too rapidly (less than 1 hr). We initially concentrated on the Bcl-2 family members, since we had tentative data indicating these proteins' involvement in sensitizing NB after drug treatment [8]. Three members of Bcl-2 family which have been described to be direct targets of Akt – Bim [29], Bax [30] and Bad [31] – were immunoprecipitated, either after 12.5 hrs of doxorubicin treatment, or after 12.5 doxorubicin/0.5 hr NVP-BEZ235 combination treatment. To assess whether one or more proteins of the Bcl-2 family changed their binding partners upon combination treatment the immunoprecipitates were probed for interactions with other phospho-Serine/Threonine containing proteins (Fig. 5A). Disappointingly, we found little differences between the phosphorylation pattern of proteins associated with these three members of Bcl-2 family after doxorubcin treatment alone or posttreatment, suggesting that modulation of Bcl-2 family members might not be a major contributing factor by which posttreatment with NVP-BEZ235 sensitizes SHEP NB cells for apoptosis. Interestingly, upon posttreatment an approximately 30 kDa protein consistently co-immunoprecipitated with each of the three Bcl-2 family proteins (Fig. 5A). We surmised that this unknown protein must be a) mitochondrial, as it associates with several members of the Bcl-2 family, b) relatively abundant, as we could detect a strong signal, c) regulated by Serine/Threonine phosphorylation, as it was detected via a phospho-specific antibody, d) regulated by the PI3K/Akt pathway, as inhibition of that pathway led to the protein's phosphorylation and e) involved in apoptosis. Thus, we identified this protein as VDAC1/Porin [32] (Fig. 5A), a 31 kDa key regulator within the mitochondrial permeability transition pore complex, which is closely associated with Bcl-2 family proteins [33].

VDAC1 phosphorylation appears to be dependent on NVP-BEZ235 (Fig. 5A). To further verify this we immunoprecipitated VDAC1 from either untreated cells, cells treated with doxorubicin or NVP-BEZ235 alone, or cells treated with the posttreatment combination (Fig. 5B). When probing the protein's phosphorylation status, we only detected Serine/Threonine phosphorylation when cells had been treated with both doxorubicin and NVP-BEZ235, suggesting that a 0.5 hr treatment with NVP-BEZ235 is not sufficient to alter the phosphorylation of cells that are not stressed by prolonged exposure to chemotherapy. The association between VDAC1 and members of the Bcl2 family seems to be unaltered, with the possible exception of Bad, further indicating that these proteins probably do not contribute to the sensitization effect observed upon posttreatment.

VDAC1 is a target of GSK3β, which in turn is negatively regulated by Akt, i.e. inhibition of Akt activates GSK3β and GSK3β-mediated phosphorylation of VDAC1 is associated with increased cytotoxicity [34]. To investigate whether NVP-BEZ235 sensitizes SHEP NB cells for apoptosis during posttreatment via altering GSK3β activity, we first analyzed GSK3β phosphorylation (Fig. 5C). While 0.5 hr NVP-BEZ235 seems to have little effect on GSK3β phosphorylation, prolonged exposure to doxorubicin leads to an enhanced GSK3β phosphorylation, suggesting increased PI3K/Akt signaling activity. This is in line with previous data that show doxorubicin to be an activator of the PI3K/Akt pathway [35], [36], [37]. Next we immunoprecipitated GSK3β from cells treated with doxorubicin or cells treated with the posttreatment combination (Fig. 5D). Crucially, GSK3β was only found to be associated with VDAC1 in the presence of doxorubicin and NVP-BEZ235. To show the functional relevance of this interaction we used SB415286, an inhibitor of GSK3, which not only disrupts the interaction of GSK3β and VDAC1 (Fig. 5E), but also counteracts the apoptosis-sensitizing effect of posttreatment (Fig. 5F). Thus we mapped the underlying mechanism responsible for the sensitizing effect of the posttreatment (i.e. the application of NVP-BEZ235 after initiation of doxorubicin treatment) to the level of the mitochondria. More precisely, inhibition of the PI3K/Akt signaling cascade activates GSK3β, which in turn leads to VDAC1 phosphorylation. Phosphorylated VDAC1 is an integral part of the mitochondrial permeability transition (PT) pore complex [33]. It is a key player of mitochondrial apoptosis [32], mediating the release of cytochrome *c* into the cytosol and thus causing caspase-3 activation [38].

**Figure 6:**
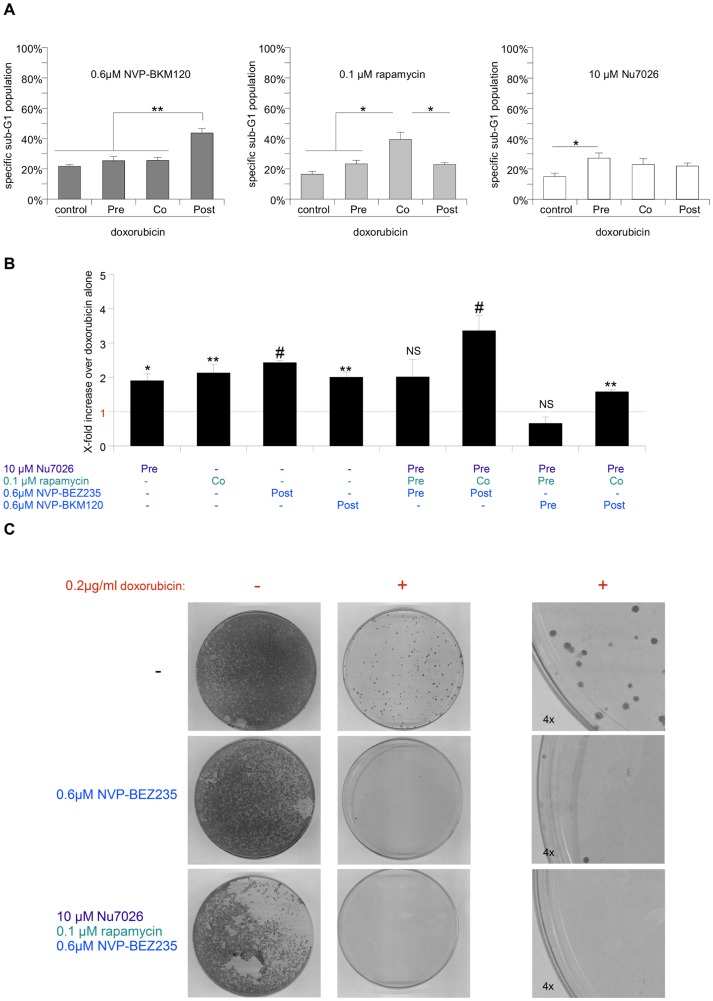
The effect of several inhibitors of PI3K/mTOR signaling on SHEP NB cell survival. **A** SHEP NB cells were treated with doxorubicin for 24(control), or in the presence of the indicated pharmacological inhibitor, which was given 12 hrs prior to doxorubicin (Pre), or simultaneously with doxorubicin (Co), or 12 hrs after doxorubicin (Post). Apoptosis was determined by FACS analysis of the DNA fragmentation of propidium iodide-stained nuclei. **B** Comparison of different treatment strategies, either using the pharmacolgical inhibitors as single agents or in combination. The sensitization effect is depicted as X-fold increase in cell death (as determined by FACS analysis of the DNA fragmentation of propidium iodide-stained nuclei) over treatment with doxorubicin alone. **C** Cells were either left untreated or treated with either NVP-BEZ235 posttreatment, or the complex combination therapy shown to work best in B, in the presence or absence of doxorubicin. Cells were treated with Nu7026 for 24.5 hrs, with doxorubicin and/or rapamycin for 12.5 hrs, 0.5 hrs with NVP-BEZ235 and allowed to grow 10 days. In A and B mean+s.e.m. values of three independent experiments carried out in triplicate are shown, in C a representative result of two independent experiments is depicted. Statistical analysis was carried out by two-sided Student's *t*-test; * P-value <0.01; ** P-value <0.001; # P-value <0.0001.

Finally, to confirm that it is indeed the inhibition of PI3K and not of mTOR that mainly mediates apoptosis sensitization (via modulation of the GSK3β/VDAC1 arm of the signaling network), we used three additional pharmacological inhibitors of the PI3K/Akt signaling network and tested their efficacy in a similar pre/co/posttreatment schedule. The inhibitors selected were the aforementioned NVP-BKM120, which only targets PI3K, the mTOR-specific inhibitor Rapamycin, as well as the DNA-PK-specific inhibitor Nu7026, which we previously identified as a main component in sensitizing Glioblastoma for apoptosis [17]. In accordance with our proposed model, inhibition of PI3K by NVP-BKM120 was not only the most effective of the three substances tested, but also was most potent during posttreatment (Fig. 6A). In contrast, inhibition of mTOR predominantly sensitizes cells when administered concurrently with doxorubicin (Fig. 6A). Interestingly, blocking DNA-PK activity by Nu7026 is only mildly efficient when used as a pretreatment component for doxorubicin-induced apoptosis (Fig. 6A). Based on these findings we designed a complex combination therapy consisting of Nu7026 pretreatment, Rapamycin cotreatment with doxorubicin, followed by NVP-BEZ235 posttreatment, which further increases the sensitization effect compared to single treatments and maximal inhibition by all three inhibitors. This combination not only significantly increases DNA fragmentation (Fig. 6B, red line indicates effect of doxorubicin as single agent), but also shows a striking effect in long term culture experiments, even in the absence of a chemotherapeutic agent (Fig. 6C). In contrast treatment with NVP-BEZ235 has little long term effect on the cells, but also leads to a potent reduction in cell numbers when used in posttreatment combined with doxorubicin (Fig. 6C). The precise relevant targets which contribute to the enhanced chemosensitization observed after application of complex combination therapy (Fig. 6B & C) remain to be fully elucidated. While we present data that is consistent with the model that post-treatment with NVP-BEZ235 or NVP-BKM120 is mainly due to modulation of VDAC1 phosphorylation, pre-treatment with Nu7026 most likely prevents DNA-PK-mediated repair of doxorubicin-induced DNA damage [17]. Interestingly, rapamycin mainly sensitizes SHEP NB cells when administered as co-treatment, suggesting a more complex relationship between chemotherapeutic agent and inhibitor. Prolonged exposure to rapamycin does not sensitize cells anymore, possibly due to metabolic modulation and autophagy induction [26]. Short term exposure, although sufficient to block mTOR activity, as assessed by S6 phosphorylation, is also not sufficient to sensitize cells for apoptosis. Further experiment will be needed to clarify which downstream targets of mTOR mediate this sensitization.

Taken together these data suggest that while PI3K/Akt/GSK3β/VDAC1 signaling is the most dominant in NB cells, additional PI3K-dependent targets can also be inhibited in a complex combination therapy that further enhances cell death. As with simple combination therapy, the sequential dosing during the complex combination therapy is essential.

### Concluding remarks

In this study we show that NB cells can be sensitized for apoptosis by combination treatment of chemotherapy, such as doxorubicin, and NVP-BEZ235. Importantly, this inhibition can be altered in efficacy by modulating the temporal relation between the two substances given, administering the inhibitor prior to the chemotherapeutic desensitizes cells for death compared to concurrent treatment, while application of NVP-BEZ235 12 hrs after initiation of doxorubicin treatment significantly enhances the antitumorigenic potential of this combination. Furthermore, these findings are not only of general importance in tumor therapy, as we could also validate our findings in GBM, but are also in line with recent reports indicating that a brief, strong inhibition of survival signals is equally potent as a strong, prolonged blockage of those signaling cascades [14].

Our data show that brief treatment with a pharmacological inhibitor after treatment with DNA-damaging chemotherapy is often preferable to prolonged treatment. While other recent studies have indicated the potential of combining conventional therapy with pharmacological modulators of the PI3K/Akt signaling cascade, such as NVP-BEZ235 [39], [40] and other small molecule inhibitors [8], [41], the question of timing has – to our knowledge - so far just begun to be addressed [13]. Here, we present data which would be consistent with a model suggesting that a strong, prolonged inhibition of the PI3K/Akt network does not optimally sensitize NB cells for increased apoptosis, a process that is after all dependent on energy consumption and therefore on metabolic activity [41], which is – at least in part – regulated by PI3K/Akt signaling [42].

It is possible that additional alterations in the cells occur upon prolonged NVP-BEZ235 treatment which counteract the sensitization effect despite continuous inhibition of GSK3β. One such alteration is the induction of a pronounced G1 arrest upon prolonged preincubation, as we have documented in Figure 1. This is in line with previous findings indicating that NVP-BEZ235 causes a cell cycle arrest [7], [43] and thus protects cells from cell cycle-dependent drugs. However, although doxorubicin is frequently classified as a cell cycle-dependent topoisomerase IIα poison [44], [45], our own data (Fig. S3D), as well as a growing body of evidence suggest that it is also a potent inducer of cell death in a cell cycle-independent manner [46], [43], [47], [48]. The antiproliferative effect of NVP-BEZ235 is also seen in multiple myeloma cells, where moderate preincubation of with NVP-BEZ235 leads to an increase in doxorubicin-induced apoptosis [43]. An alternative explanation for the decreased cell death observed upon preincubation with the PI3K/mTOR inhibitor might be that doxorubicin-induced apoptosis is inhibited by increased autophagy which can be observed after NVP-BEZ235 treatment. Interestingly, doxorubicin has been previously shown to interact with autophagy mediating proteins [46]. This would be in line with previous reports suggesting that sequential application of drugs can achieve a sensitization to doxorubcin via rewiring apoptotic signaling networks and independently of the cell cycle [13].

The implications of autophagic processes in cell death are still strongly debated. The idea that cells are exclusively dying by reaching a certain threshold of self-destruction is still controversial, since cell death with autophagic features always coexists with morphological properties of other cell death types within the same cell [49]. It is more likely that autophagy maintains cell viability through production of valuable metabolites, even during excessive autophagy-induction caused by PI3K inhibitor treatment. Therefore, it is tempting to speculate that the failure of NVP-BEZ235 pretreatment to sensitize cells for apoptosis, or – indeed – in several cases to even exhibit a desensitizing effect, is due to the increased induction of autophagy. However, further experiments are needed to clarify this point.

The use of NVP-BEZ235 in post-treatment is not without controversy, as chemotherapeutic agents – and doxorubicin in particular – have been shown to be activators of PI3K/Akt signaling in a wide variety of malignancies [35], such as myelogenous leukemia [36] and breast cancer [37]. If the pharmacological inhibitor is only added after complex signaling cascades have already been transcriptionally activated by doxorubicin, inhibition of relative upstream mediators, such as PI3K or mTOR, is likely to be of reduced relevance. Here we show that in NB cells, and to a lesser extent in GBM, activation of PI3K/Akt signaling during post-treatment is mainly mediated via continuous Akt/GSK3β/VDAC1 phosphorylation and therefore amenable to a short, potent inhibition. Further research is needed to investigate whether in other types of cancerous growth alternative protocols, either with altered timing or additional pharmacological inhibitors, might be more promising.

Furthermore, our findings show that precisely timed inhibitions of what is generally thought of as a single pathway can further potentiate the therapeutic efficacy. Importantly, the PI3K/Akt/mTOR signaling cascade is not a linear pathway, but a complex cascade that can initiate multiple parallel arms concurrently, which are not only involved in cellular survival, but also in multiple other aspects of cellular behavior, such as metabolism, proliferation, motility and autophagy [50]. Depending on the precise timing of inhibition, a single pharmacological inhibitor can have different indirect targets, that might antagonize or synergize each other. *Vice versa*, multiple precisely timed inhibitions of a single pathway with pharmacological inhibitors with distinct primary targets might potentiate the apoptosis sensitization even further, as shown here.

While the challenge remains to establish a clear and relevant *in vivo* model that takes into account the pharmacokinetics, modes of drug delivery and differences in human versus rodent metabolism, the further investigation into the role of the temporal relation of different therapeutic components will be of increased importance. While the current clinical paradigm is to utilize a constant high level of the pharmacological inhibitors during treatment, our findings strongly suggest that such an approach can actually antagonize the cytotoxic effect of the chemotherapeutic, and therefore the current clinical treatment protocols need to be re-evaluated.

## Supporting Information

Figure S1 **The effects of the PI3K inhibitor NVP-BKM120 on SHEP NB cells.** **A** SHEP NB cells were left untreated (control) or treated for indicated lengths of time with 0.6 µM of NVP-BKM120. Protein expression levels and phosphorylation status of Akt, S6 ribosomal protein and β-actin were analyzed by Western blotting. **B** Cells were cultured either in the presence or absence of 0.6 µM NVP-BKM120 for 24, 48 and 72 hrs, followed by FACS analysis of the DNA fragmentation of propidium iodide-stained nuclei. The percentage of absolute DNA fragmentation is shown as readout for apoptosis. **C** Cells were seeded and allowed to adhere o/n before treatment with 0.6 µM of NVP-BKM120 commenced. 24, 48 and 72 hrs after treatment total cell numbers of treated and untreated cells were determined. **D** The cell cycle distribution (control and samples treated with 0.6 µM of NVP-BKM120) was determined after indicated times by FACS analysis of propidium iodide-stained nuclei. In A a representative result of at least three independent experiments is depicted, while in B to D mean values of three independent experiments carried out in triplicate are shown (in B and C+s.e.m.). Statistical analysis was carried out by two-sided Student's *t*-test; * P-value <0.01; ** P-value <0.001; # P-value <0.0001.
(TIF)

Figure S2 **The effects of the mTOR inhibitor rapamycin on SHEP NB cells.** **A** SHEP NB cells were treated for 24 h with the solvent dimethylsulfoxide (control), or for indicated lengths of time with 0.1 µM of rapamycin. Protein expression levels and phosphorylation status of Akt, S6 ribosomal protein and β-actin were analyzed by Western blotting. **B** Cells were cultured either in the presence or absence of 0.1 µM of rapamycin for 24, 48 and 72 hrs, followed by FACS analysis of the DNA fragmentation of propidium iodide-stained nuclei. The percentage of absolute DNA fragmentation is shown as readout for apoptosis. **C** Cells were seeded and allowed to adhere o/n before treatment with 0.1 µM of rapamycin commenced. 24, 48 and 72 hrs after treatment total cell numbers of treated and untreated cells were determined. **D** The cell cycle distribution (control and samples treated with 0.1 µM of rapamycin) was determined after indicated times by FACS analysis of propidium iodide-stained nuclei. In A a representative result of at least three independent experiments is depicted, while in B to D mean values of three independent experiments carried out in triplicate are shown (in B and C+s.e.m.). Statistical analysis was carried out by two-sided Student's *t*-test; * P-value <0.01; ** P-value <0.001; # P-value <0.0001.
(TIF)

Figure S3 **The effects of doxorubicin on SHEP NB cells.** **A** Determination of the cell cycle distribution of SHEP NB cells (untreated control and samples treated with 0.2 µg/ml doxorubicin) after indicated times by FACS analysis of propidium iodide-stained nuclei. **B** The kinetic of effector caspase 3 processing after treatment with 0.2 µg/ml doxorubicin was determined via Western blot analysis, β-actin served as loading control. **C** Cells were either left untreated or treated with 0.2 µg/ml doxorubicin for 12 hrs, followed by apoptosis determination via FACS analysis of the DNA fragmentation of propidium iodide-stained nuclei. **D** Cells were either left untreated or treated with 0.2 µg/ml doxorubicin for 24 hrs. To analyze the cell cycle phase-dependent cell death, cells were stained with Hoechst 33258 dye. The diagram shows an exemplary result, while the table summarizes all data sets. Figures in red indicate the percentage of dead cells per cell cycle phase. **E** Cells were either left untreated, or treated with NVP-BEZ235 for the indicated lengths of time. Conversion of LC3-I to LC3-II was analyzed by Western blotting, β-actin served as loading control. In A, C and D mean+s.e.m. of three independent experiments carried out in triplicate are shown, in B and E a representative blot of two independent experiments is shown. Statistical analysis was carried out by two-sided Student's *t*-test; * P-value <0.01; ** P-value <0.001; # P-value <0.0001.
(TIF)

File S1 **Supporting Information Materials & Methods.**
(PDF)
