# Supplementary figures and images for: Sequential Dosing in Chemosensitization: Targeting the PI3K/Akt/mTOR Pathway in Neuroblastoma
Source: PLoS One. 2013 Dec 31;8(12):e83128. doi: 10.1371/journal.pone.0083128 (PMC3877010; doi:10.1371/journal.pone.0083128)

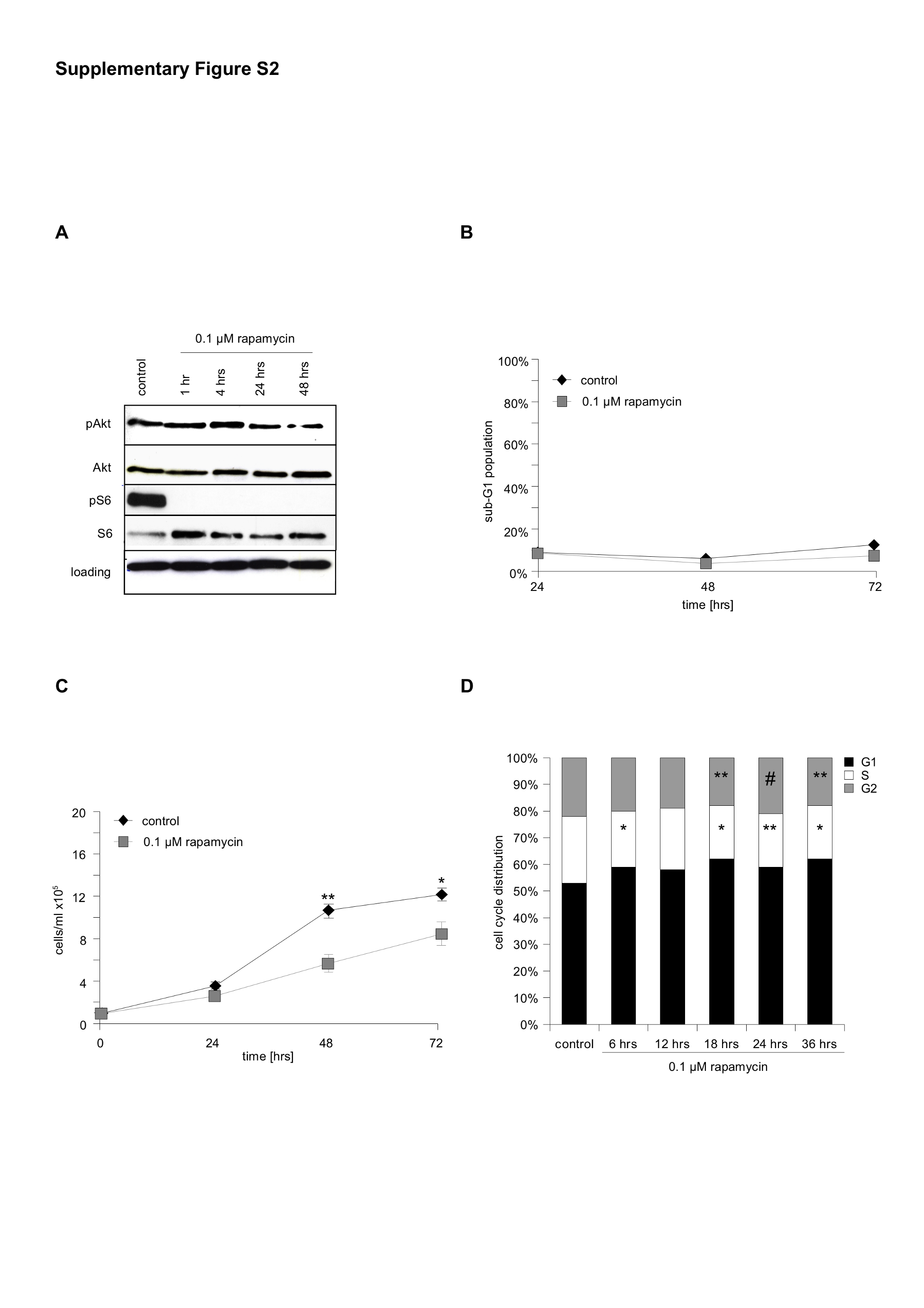

Supplement: Figure S2 — The effects of the mTOR inhibitor rapamycin on SHEP NB cells. A SHEP NB cells were treated for 24 h with the solvent dimethylsulfoxide (control), or for indicated lengths of time with 0.1 µM of rapamycin. Protein expression levels and phosphorylation status of Akt, S6 ribosomal protein and β-actin were analyzed by Western blotting. B Cells were cultured either in the presence or absence of 0.1 µM of rapamycin for 24, 48 and 72 hrs, followed by FACS analysis of the DNA fragmentation of propidium iodide-stained nuclei. The percentage of absolute DNA fragmentation is shown as readout for apoptosis. C Cells were seeded and allowed to adhere o/n before treatment with 0.1 µM of rapamycin commenced. 24, 48 and 72 hrs after treatment total cell numbers of treated and untreated cells were determined. D The cell cycle distribution (control and samples treated with 0.1 µM of rapamycin) was determined after indicated times by FACS analysis of propidium iodide-stained nuclei. In A a representative result of at least three independent experiments is depicted, while in B to D mean values of three independent experiments carried out in triplicate are shown (in B and C+s.e.m.). Statistical analysis was carried out by two-sided Student's t-test; * P-value <0.01; ** P-value <0.001; # P-value <0.0001. (TIF) [file pone.0083128.s002.tif]

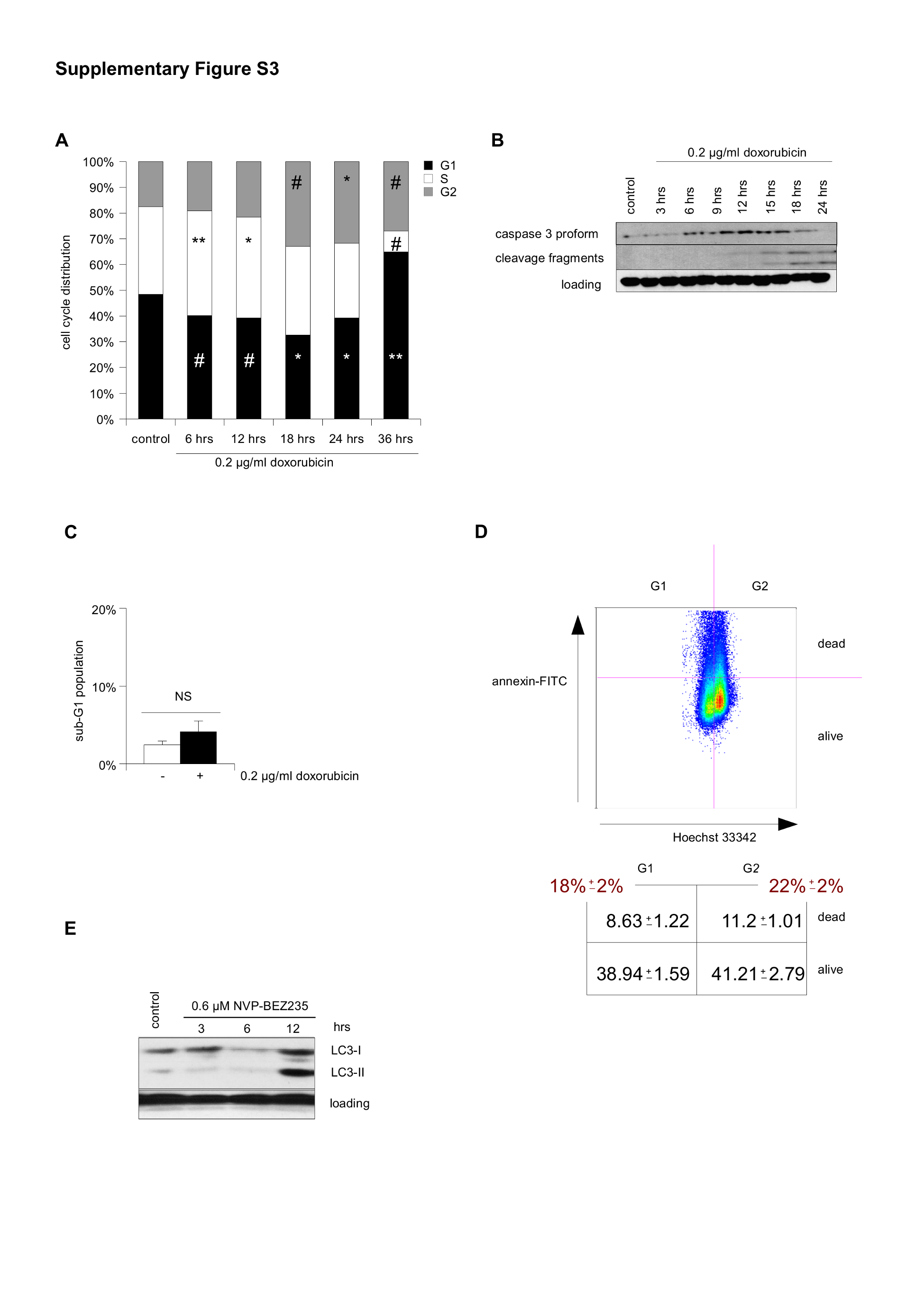

Supplement: Figure S3 — The effects of doxorubicin on SHEP NB cells. A Determination of the cell cycle distribution of SHEP NB cells (untreated control and samples treated with 0.2 µg/ml doxorubicin) after indicated times by FACS analysis of propidium iodide-stained nuclei. B The kinetic of effector caspase 3 processing after treatment with 0.2 µg/ml doxorubicin was determined via Western blot analysis, β-actin served as loading control. C Cells were either left untreated or treated with 0.2 µg/ml doxorubicin for 12 hrs, followed by apoptosis determination via FACS analysis of the DNA fragmentation of propidium iodide-stained nuclei. D Cells were either left untreated or treated with 0.2 µg/ml doxorubicin for 24 hrs. To analyze the cell cycle phase-dependent cell death, cells were stained with Hoechst 33258 dye. The diagram shows an exemplary result, while the table summarizes all data sets. Figures in red indicate the percentage of dead cells per cell cycle phase. E Cells were either left untreated, or treated with NVP-BEZ235 for the indicated lengths of time. Conversion of LC3-I to LC3-II was analyzed by Western blotting, β-actin served as loading control. In A, C and D mean+s.e.m. of three independent experiments carried out in triplicate are shown, in B and E a representative blot of two independent experiments is shown. Statistical analysis was carried out by two-sided Student's t-test; * P-value <0.01; ** P-value <0.001; # P-value <0.0001. (TIF) [file pone.0083128.s003.tif]
