# Supplementary material for: Sequential Dosing in Chemosensitization: Targeting the PI3K/Akt/mTOR Pathway in Neuroblastoma
Source: PLoS One. 2013 Dec 31;8(12):e83128. doi: 10.1371/journal.pone.0083128 (PMC3877010; doi:10.1371/journal.pone.0083128)
Supplement: File S1 — Supporting Information Materials & Methods. (PDF) [file pone.0083128.s004.pdf]

Supplementary materials & methods

Cell cycle phase-dependent death

To analyze the cell cycle phase-dependent cell death, 5 µg/ml of Hoechst 33258 stain (Sigma-Aldrich) was added to the medium for 10 min. Then cells were treated with trypsin and washed twice in Annexin buffer (Sterofundin solution with 1% Hepes), followed by a 30 min incubation with an AnnexinV-FITC conjugate (Invitrogen® Life Technologies, Darmstadt, Germany). Cells were then analyzed by fluorescence-activated cell-sorting (LSRII, BD Bioscience, Heidelberg, Germany) and statistical evaluation was performed using *Flow Jo* software (Tree Star, Inc., OR, USA).
